# Disseminated Dermal Leishmaniasis Caused by *Leishmania siamensis* In a Systemic Steroid Therapy Patient

**DOI:** 10.4269/ajtmh.13-0711

**Published:** 2014-11-05

**Authors:** Nopadon Noppakun, Kanyarat Kraivichian, Padet Siriyasatien

**Affiliations:** Division of Dermatology, Department of Medicine, Faculty of Medicine, Chulalongkorn University, Bangkok 10330 Thailand; Bumrungrad International Hospital, Bangkok 10110 Thailand; Department of Parasitology, Faculty of Medicine, Chulalongkorn University, Bangkok 10330 Thailand; Excellence Center for Emerging Infectious Diseases, King Chulalongkorn Memorial Hospital, Thai Red Cross Society, Bangkok 10330 Thailand

## Abstract

*Leishmania siamensis* infection was recently reported from Thailand. Clinical presentation of *L. siamensis* infections is generally related to human immunodeficiency virus infection. Herein, disseminated dermal *L. siamensis* infection in a systemic steroid therapy patient from Myanmar is described.

A 60-year-old male Burmese living in Yangon had never traveled abroad. He developed fever, multiple infiltrative erythematous nodules on his body 3 months before coming to Thailand. He visited a physician in Yangon and was diagnosed as Sweet's syndrome. The patient was treated with prednisolone for 2 months without improvement.

Repeat biopsy showed diffuse histiocytic infiltrate and multinucleated giant cells in the upper and deep dermis. Many rounds to oval small organisms were present in histiocytes and fibrous stroma. He was diagnosed as histoplasmosis and was treated with itraconazole, his general condition was improving, however the cutaneous lesions worsened (Figure 1A and B). Laboratory testing for antinuclear antibodies, anti-human immunodeficiency virus (HIV), C-reactive protein were negative or within normal limits.

**Figure 1.**
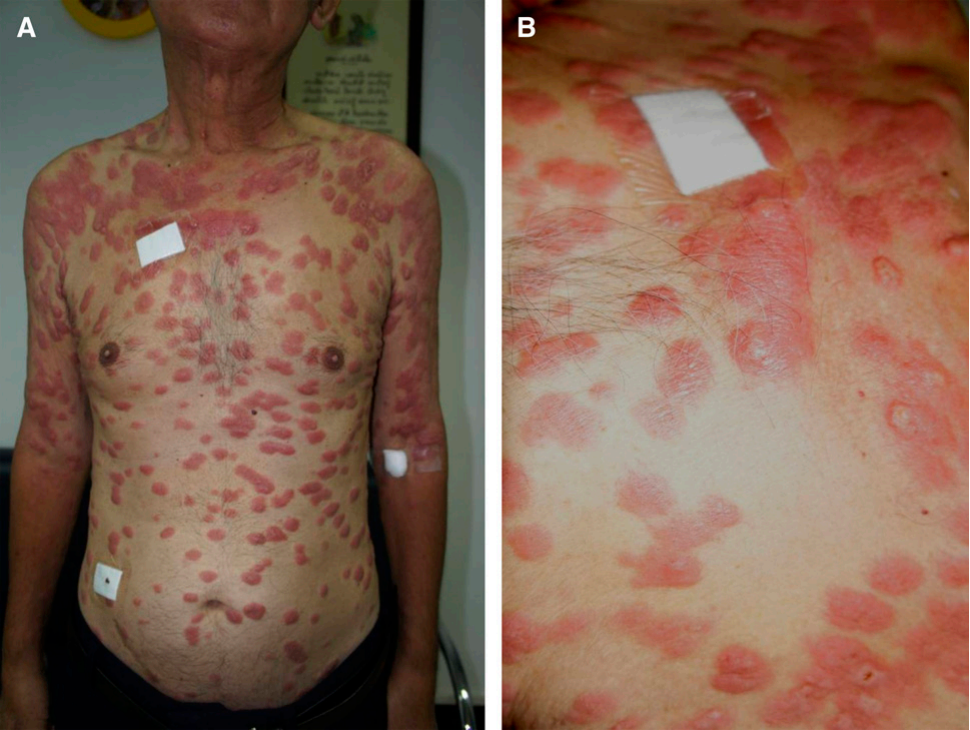
Disseminated dermal leishmaniasis caused by *Leishmania siamensis*. Multiple erythematous, shiny infiltrative plaques and nodule on face, trunk, and extremities were observed (**A**). Infiltrative erythematous nodules, ulcers were observed on some nodules (**B**).

New skin biopsy showed numerous small yeast-liked organisms, and some of these organisms were contained small basophilic dots in the cytoplasm opposite to nuclei (Figure 2). This finding suggested that it was *Leishmania*. Polymerase chain reaction (PCR) using a primer set annealed specifically to the 18S rRNA gene of the internal transcribed spacer1 (ITS1) of *Leishmania* sp. *Leishmania siamensis* was identified by nucleotide sequencing of the PCR products compared with sequences of *L. siamensis* previously reported (accession no. JQ001751) and has showed to be 100% identical.^1^ All specimens used to culture for *Leishmania* were negative.

**Figure 2.**
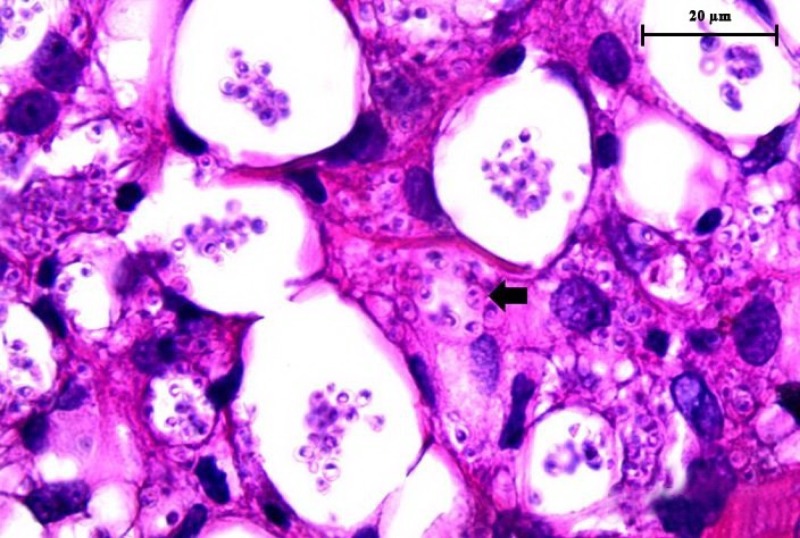
Histopathological examination of the skin biopsy from the patient, hematoxylin and eosin (H&E) staining reveals numerous intracellular organisms containing nucleus and bar-shaped kinetoplast (arrow). This indicated amastigotes of *Leishmania* parasites infection (100×).

After a final diagnosis of disseminated dermal leishmaniasis was established, the patient was treated with intravenous amphotericin B 60 mg per day for 40 days. His cutaneous lesions markedly regressed. He was discharged without any medication. When he returned for follow-up 2 months later, he reported that he had gained weight and appetite. Few skin lesions still remained but they were flattened and decreased in size.

Leishmaniasis has been described as an opportunistic infection in immunocompromised patients. Leishmaniasis in glucocorticoides-treated patients has been reported in three cases infected with *Leishmania infantum*.^2^ *Leishmania siamensis* infection was described mostly in acquired immunodeficiency syndrome (AIDS) patients from Thailand. This is the first autochthonous leishmaniasis caused by this novel species in Myanmar.
